# Supplementary material for: Using GDACS to anticipate clinical and operational burden after earthquakes: A global event-level analysis (2020–2024)
Source: PLoS One. 2026 Jan 13;21(1):e0339018. doi: 10.1371/journal.pone.0339018 (PMC12798983; doi:10.1371/journal.pone.0339018)
Supplement: S1 Table — Event-level sensitivity analyses for the association between GDACS indicators and outcomes across 85 composite earthquake events (2020–2024). Each row represents a pre-specified robustness check: exclusion of the February 2023 Türkiye sequence, use of exposure-normalized mortality (deaths per 100,000 exposed), comparison of deaths between Red and Orange alerts, and alternative deployment definitions in logistic models (any temporary facility vs field hospital only). Columns report the number of events (N), the effect metric (Spearman’s ρ, Mann–Whitney U, or odds ratio per 1-point increase in GDACS score), point estimates with 95% confidence intervals where applicable, p values, Benjamini–Hochberg–adjusted q values, and brief interpretive notes. GDACS = Global Disaster Alert and Coordination System; OR = odds ratio; CI = confidence interval; ML = maximum likelihood. (DOCX) [file pone.0339018.s001.docx]

**S1 Table. Sensitivity Analyses**

| **Analysis** | **N** | **Metric** | **Estimate** | **95% CI** | **p** | **q** | **Note** |
| --- | --- | --- | --- | --- | --- | --- | --- |
| Exclude Türkiye Feb-2023 sequence | 83 | Spearman ρ (Score vs Deaths) | 0.495 | – | 1.93×10⁻⁶ | 1.93×10⁻⁶ | Robust to removal of Feb‑2023 Türkiye shocks |
| Exposure-normalized endpoint (Deaths/100k) | 19 | Spearman ρ (Score vs Deaths/100k) | 0.501 | – | 0.0289 | 0.0289 | Exploratory (small n; Red=1, Orange=18) |
| Red vs Orange (Deaths) | 85 | Mann–Whitney U | U=973 | – | 6.36×10⁻⁶ | 6.36×10⁻⁶ | Cliff’s δ=0.683 (large) |
| Logistic (ML) — Any temporary facility | 85 | OR per +1 GDACS | 4.04 | (1.79–9.08) | 7.41×10⁻⁴ | 7.41×10⁻⁴ | Sensitivity (primary in text = Firth) |
| Logistic (ML) — Field hospital | 85 | OR per +1 GDACS | 42.72 | (4.73–385.71) | 8.24×10⁻⁴ | 8.24×10⁻⁴ | Near-separation; very strong gradient |
