# Supplementary material for: Using GDACS to anticipate clinical and operational burden after earthquakes: A global event-level analysis (2020–2024)
Source: PLoS One. 2026 Jan 13;21(1):e0339018. doi: 10.1371/journal.pone.0339018 (PMC12798983; doi:10.1371/journal.pone.0339018)
Supplement: S2 Table — Panel A summarises apparent discrimination and overall accuracy for maximum-likelihood logistic models predicting field-hospital deployment and any temporary facility deployment from the GDACS composite score (N = 85 composite events, 2020–2024). Reported statistics include the Hosmer–Lemeshow goodness-of-fit χ² test (degrees of freedom, p value), the area under the receiver-operating characteristic curve (AUC), and the Brier score; calibration slope and intercept are derived from same-sample recalibration (apparent calibration). External validation was not performed. Panel B reports variable-wise missingness counts in the analytic dataset (N = 85), indicating complete availability of core GDACS and outcome variables and partial availability of GDACS-modelled exposure within 100 km. GDACS = Global Disaster Alert and Coordination System; AUC = area under the ROC curve. (DOCX) [file pone.0339018.s002.docx]

**S2 Table. Model diagnostics and missingness**

**Panel A. Apparent calibration & discrimination (ML fits)**

| Model | Outcome | N | Hosmer–Lemeshow χ² (df, p) | AUC | Brier |
| --- | --- | --- | --- | --- | --- |
| A | Field hospital | 85 | 1.022 (6, 0.985) | 0.982 | 0.0336 |
| A | Any temporary facility | 85 | 5.601 (6, 0.469) | 0.739 | 0.1625 |

*Note: Calibration slope/intercept from same-sample recalibration equal ~1.00/0.00 by identity (apparent calibration). External validation not performed.*

**Panel B. Missingness counts (rep=85)**

| Variable | Missing (n) |
| --- | --- |
| Magnitude (Mw) | 0 |
| Depth (km) | 0 |
| GDACS Score | 0 |
| Deaths (count) | 0 |
| Field hospital deployment | 0 |
| Any temporary facility deployment | 0 |
| Exposure within 100 km | 66 |
